# Dietary high-purity tribromoethanol (TBE) supplementation reduces enteric methane emissions in beef cattle

**DOI:** 10.1093/tas/txag074

**Published:** 2026-05-29

**Authors:** Robin Malik, John-Fredy Ramirez-Agudelo, Ermias Kebreab

**Affiliations:** Department of Animal Science, University of California, Davis, CA, 95616, United States; Department of Animal Science, University of California, Davis, CA, 95616, United States; Department of Animal Science, University of California, Davis, CA, 95616, United States

**Keywords:** cattle, climate change, greenhouse gas emissions, methane, tribromoethanol

## Abstract

The livestock industry contributes substantially to global greenhouse gas emissions, with enteric methane (CH_4_) from ruminants representing a major source. Feed additives that suppress methanogenesis have emerged as promising strategies, provided they do not compromise animal performance. The objective of this study was to evaluate the effects of high-purity 2,2,2-tribromoethanol (TBE) on enteric gas emissions (CH_4_, CO_2_, H_2_), dry matter intake (DMI), average daily gain (ADG), and feed efficiency (FE, ADG/DMI) in beef steers. Twenty-four Red Angus steers (initial body weight (BW) = 477 ± 27 kg) were blocked by BW and randomly assigned to one of three dietary treatments (*n* = 8 per treatment group): control (0 mg TBE/kg DM), low (15 mg TBE/kg DM), or high (30 mg TBE/kg DM). The TBE was dissolved in canola oil, mixed into a premix, and incorporated into the total mixed ration formulated to meet or exceed National Academies of Sciences, Engineering, and Medicine requirements for finishing beef cattle (ADG, 1.36–1.81 kg/d). The experiment followed a replicated 3 × 3 Latin square design with three 28-d experimental periods, during which treatments were rotated so that each animal received each treatment once. Enteric gas emissions were measured using a GreenFeed system across 8 sampling time points over a 3-day period during the final week of each experimental period. Individual DMI was recorded daily from weighed feed offered and refusals, and BW was measured weekly. Statistical analyses were performed using linear mixed-effects models with treatment and period as fixed effects and animal nested within square as random effects in a repeated measures analysis. Daily TBE supplementation reduced (*P* < 0.001) CH_4_ production (g/d) by 15.6% and 32.2% for the low and high treatment groups, respectively, relative to control. Methane yield (g/kg DMI) and intensity (g/kg ADG) were also reduced (*P* < 0.001 and *P* = 0.005, respectively) by 10.7% (low) and 28.0% (high), and 12.8% (low) and 33.4% (high), respectively. DMI, ADG, and FE did not differ among treatments. These findings suggest that TBE supplementation can effectively reduce enteric CH_4_ emissions in beef cattle without adversely affecting feed intake, growth performance, or feed efficiency. Tribromoethanol may therefore represent a potential CH_4_ mitigation strategy warranting further evaluation regarding long-term efficacy, safety, and practical application in beef production systems.

## Teaser

Supplementing beef cattle diets with high-purity tribromoethanol reduced daily methane emissions by up to 32% without affecting feed intake, or weight gain, offering a promising strategy for climate-smart beef production.

## Introduction

Climate change and food security are closely linked global challenges that pose considerable risks to environmental stability, economic systems, and human well-being (Fanzo et al. 2025). Livestock production plays an essential role in global food systems by providing high-quality protein and micronutrients that are difficult to replace in many regions of the world (Smith et al. 2013). At the same time, the livestock sector has come under increasing scrutiny because of its contribution to greenhouse gas emissions, particularly methane (CH_4_), carbon dioxide (CO_2_), and nitrous oxide (Sejian et al. 2015). Balancing the nutritional and socioeconomic benefits of livestock with the need to reduce environmental impacts remains a central challenge for sustainable food production.

Global production of animal-sourced foods is estimated to generate approximately 6.2 Gt CO_2_ equivalents annually, representing around 12% of total anthropogenic greenhouse gas emissions (FAO 2023). Cattle account for the largest share of livestock greenhouse gas emissions due to their widespread distribution, population size, and high feed intake, contributing approximately 3.8 Gt CO_2_ equivalents per year, or roughly 62% of all livestock emissions (Cheng et al. 2022; FAO 2023). Enteric CH_4_ represents approximately 46% of all livestock-related greenhouse gas emissions (FAO 2023), making it the dominant emission source within the sector and a primary target for mitigation efforts.

Enteric CH_4_ is produced in the rumen as a natural by-product of microbial fermentation of mostly dietary carbohydrates (Min et al. 2022). The rumen hosts a complex community of anaerobic microorganisms in which bacteria, protozoa, fungi, and archaea interact to degrade feedstuffs and generate volatile fatty acids, including acetate, propionate, and butyrate, which serve as the main energy sources for the host animal (Cammack et al. 2018). During this process, metabolic hydrogen (H_2_) and CO_2_ are also produced as by-products (Greening et al. 2019). Elevated levels of H_2_ can inhibit specific microbial pathways under certain rumen conditions, though this does not necessarily affect overall animal performance or digestibility (Ungerfeld et al. 2022). Therefore, methanogens serve as the primary H_2_ sink in the rumen by reducing CO_2_ to CH_4_ through a multi-enzyme pathway, with the terminal reaction catalyzed by methyl-coenzyme M reductase (MCR) (Hook et al. 2010; Duin et al. 2016; Evans et al. 2019). While this pathway maintains efficient rumen fermentation (Hook et al. 2010), it also represents an energetic loss to the animal (Johnson and Johnson 1995) and a significant source of greenhouse gas emissions (Hristov et al. 2013; Knapp et al. 2014).

A wide range of strategies to mitigate enteric CH_4_ emissions have been investigated, targeting different aspects of rumen function and livestock production systems (Beauchemin et al. 2022). Nutritional strategies such as altering forage-to-concentrate ratios, lipid supplementation, and inclusion of specific feed additives can modify ruminal fermentation pathways and reduce CH_4_ per unit of feed intake (Fouts et al. 2022). More direct approaches include the use of rumen-targeted inhibitors, such as 3-nitrooxypropanol (3-NOP) (Jayanegara et al. 2018; Kim et al. 2019) and macroalgae containing halogenated compounds, particularly bromoform (Lean et al. 2021; Kebreab et al. 2025), which suppress methanogenesis by inhibiting MCR. Genetic selection for low CH_4_ emitting animals also offers a long-term reduction mitigation potential, although implementation remains complex (Wall et al. 2010).

The compound 2,2,2-Tribromoethanol (TBE) was introduced into clinical practice in the early twentieth century as a rectal anesthetic for human and veterinary use and was initially considered advantageous because of its ease of synthesis and availability of reagents (Meyer and Fish, 2005). Subsequent evidence demonstrated that TBE, similar to other halogenated compounds, could induce hepatotoxic effects at anesthetic doses, leading to a decline in its medical use after the mid-twentieth century (Meyer and Fish, 2005). Despite this history, the potential effects of TBE on rumen microbial processes, particularly methanogenesis, have received little attention. Although the mode of action of TBE has not yet been fully established, recent in vitro evidence suggests that high purity TBE can substantially reduce predicted methane production without negatively affecting ruminal fermentation characteristics (Fekri et al. 2026). Considering its chemical structure, it is highly likely that TBE inhibits methanogenesis in a manner similar to other halogenated CH_4_ analogues, including bromoform, by targeting MCR and disrupting the final step of CH_4_ formation.

Therefore, the objective of this study was to evaluate the effects of dietary supplementation with high-purity TBE on enteric CH_4_ emissions, dry matter intake (DMI), and average daily gain (ADG) in beef cattle. We hypothesized that TBE would reduce CH_4_ production in a dose-dependent manner while having minimal effects on feed intake, growth performance, and feed efficiency.

## Materials and methods

This study was conducted at the University of California, Davis, feedlot research facility. All animal procedures were approved by the University of California, Davis Institutional Animal Care and Use Committee (Protocol No. 23913) and were carried out in accordance with established animal welfare guidelines.

### Animals, experimental design and treatments

Sample size was determined using power analysis for a replicated Latin square design. With three treatment groups, an expected effect size of 0.31, equivalent to a 10% reduction in CH_4_ yield for either treatment group to control from a baseline of 10 g CH_4_/kg DMI (*SD* = 1.5), significance level of 0.05, and statistical power of 0.90, a total of 24 animals for the complete replicated 3 × 3 Latin square design was required. Given the absence of prior in vivo data for TBE, the selected inclusion rates were informed by previous in vitro work demonstrating CH_4_ inhibition at similar concentrations (Fekri et al. 2026).

Twenty-four Red Angus beef steers (average initial body weight (BW) of 477 ± 27 kg) were sourced from a single ranch in Reedley, CA, through the Western Video Market (Cottonwood, CA, USA) at approximately 10 months of age. Steers were individually housed in adjacent pens (7 m × 4 m) to facilitate controlled treatment administration and accurate monitoring of feed intake while maintaining social interaction between neighboring steers. Pens had concrete flooring bedded with rice hulls, which were refreshed weekly.

Steers were blocked by BW and randomly assigned to one of three dietary treatments: control (0 mg TBE/kg DM), low TBE (15 mg TBE/kg DM), or high TBE (30 mg TBE/kg DM), with eight steers per treatment group. Steers were assigned to a replicated 3 × 3 Latin square design consisting of three 28-d periods, such that each steer received each treatment once and each treatment occurred once per period within square. At the start of each new period, steers were reassigned evenly among the two remaining treatments to preserve Latin square balance while minimizing potential confounding associated with maintaining previous treatment groupings across periods.

The experiment was conducted over a 16-week period, consisting of a 2-week training period, a 2-week covariate period, and three 4-week experimental periods. During the training period, steers were acclimated to the GreenFeed Large Animal System (C-Lock Inc., Rapid City, SD) and habituated to individual pens. No additive was administered during training. During the covariate period, all steers were fed the control diet to establish baseline measurements and allow further acclimation to experimental conditions and measurement procedures.

### Feeding and diet preparation

Steers were fed a total mixed ration (TMR; Table 1) formulated to meet or exceed nutrient requirements for finishing beef steers according to National Academies of Sciences, Engineering, and Medicine recommendations (NASEM, 2016) (ADG, 1.36–1.81 kg/d). The basal diet remained constant across all treatments and experimental periods. Feed was offered twice daily at 7:00 am and 5:00 pm. At the start of the training period, feed was offered at 3% of BW (as-fed basis). Thereafter, steers were offered feed at 110% of their previous day’s intake. Feed refusals were collected and weighed daily prior to the morning feeding to determine individual DMI and to calculate subsequent feed allowances. Sixty percent of the daily TMR was offered in the morning and the remaining 40% in the evening. Steers had ad libitum access to clean water using valve activated livestock water bowls fitted in each individual pen.

**Table 1:** Ingredients and nutrient composition of the experimental diet.

	(% DM, unless noted)
***Ingredients***	
*** Wheat hay***	10.9
*** Rolled corn***	56.3
*** DDG***	28.7
*** Molasses***	2.26
*** Calcium carbonate***	1.52
*** Salt***	0.32
*** Magnesium Oxide***	0.03
*** Feedlot Vitamin Premix^a^***	0.04
***Nutrient Composition^b^***	
*** dry matter***	85.3
*** CP***	17.6
*** ADF (%NDF)***	10.1
*** NDF***	20.8
*** Lignin (%NDF)***	8.70
*** Ether Extract***	6.33
*** TDN***	80.5
*** Ash***	6.94
*** Calcium***	1.04
*** Phosphorus***	0.59
*** Magnesium***	0.29
*** Potassium***	0.94
*** Sodium***	0.29
*** Iron (ppm)***	119
*** Manganese (ppm)***	42.9
*** Zinc (ppm)***	89.8
*** Copper (ppm)***	18.4

DDG, dried distiller’s grain; DM, dry matter; CP, crude protein; ADF, acid detergent fiber; NDF, neutral detergent fiber; TDN, total digestible nutrient.

a Premix manufactured by Cargill Animal Nutrition (Minneapolis, MN). Contains calcium, copper, manganese, zinc, cobalt, iodine, selenium, vitamin A, vitamin D, vitamin E.

b Average over all samples taken throughout the experiment.

High-purity TBE (>99% purity; Agteria Biotech, Stockholm, Sweden) was used throughout the experiment. Because TBE was administered at very low inclusion rates, canola oil was used as a carrier to facilitate uniform incorporation into the TMR. To control for potential effects of dietary fat on CH_4_ production, the total amount of canola oil was standardized across all treatments. To achieve this, TBE was dissolved in canola oil at concentrations of 0.00%, 0.15%, and 0.30% for the control, low, and high treatments, respectively. The resulting premixes were provided at 1% of TMR DM, providing finished diets with 0, 15, and 30 mg TBE/kg, respectively. Oil solutions were prepared in bulk one week in advance to ensure complete homogenization of TBE. The required amount of oil solution was weighed for each steer prior to feeding and thoroughly mixed into the TMR by hand to ensure uniform distribution.

### Sample collection and analysis

Body weight was measured weekly using a hydraulic squeeze chute with a scale (Silencer Ranch Model, Dubas Equipment Stapleton, NE, USA). Average daily gain (kg/d) was calculated for each animal as the slope of the linear regression of BW (kg) on day of experimental period, using weekly weight measurements. Outliers were identified and removed per animal based on residuals from a global (all-period) regression of BW across all periods, applying the 1.5 × interquartile range criterion. Per-period slopes were then re-estimated on the retained observations.

Weekly samples of the TMR and feed refusals (orts) were collected and analyzed for nutrient composition, including crude protein, neutral detergent fiber, ether extract, and mineral content (Table 1). Feed samples were analyzed by Cumberland Valley Analytical Services (Waynesboro, PA) using standard AOAC International procedures for DM (AOAC 930.15), ash (AOAC 942.05), CP (AOAC 990.03), ether extract (AOAC 2003.05), and fiber fractions including NDF (AOAC 2002.04) and ADF (AOAC 973.18).

Gas emissions (CH_4_, CO_2_, and H_2_) were measured using the GreenFeed Large Animal System during the last three days of the covariate period and each experimental period. To account for diurnal variation (Hegarty, 2013), gas measurements were distributed across multiple time points. To mimic a 24-hour emission cycle, gas emission samples were recorded 8 times within a 45-hour period. Sampling times were as follows: 9:00 am, 3:00 pm, and 9:00 pm (sampling day 1), 3:00 am, 12:00 pm, and 6:00 pm (sampling day 2), and 12:00 am, and 6:00 am (sampling day 3). As steers did not have free access to the GreenFeed system, they were individually guided to the unit, and each steer was allowed up to 6 minutes to voluntarily use the system, before they were ushered back to their home pens. The GreenFeed system dispensed up to ten 30-g drops of alfalfa pellets at 20-s intervals to encourage head placement within the unit. Steers consumed an average of 138 g ± 15.6 g (DMI) of alfalfa pellets during each 6-minute visitation window. The GreenFeed system was auto-calibrated every 6 days using a gas mixture with known concentrations of CH_4_ (3.5% by volume) and CO_2_ (11.5% by volume) through a precision flow controller at a fixed rate (10 L/min), while the GreenFeed unit drew in ambient air at approximately 40 L/s. Additional CO_2_ recovery calibrations were performed a week prior to each GreenFeed sampling period, with mean recoveries of 99% ± 4%.

GreenFeed records were screened before analysis using three quality-control filters. First, individual visits with a head-in-hood duration shorter than 2 min were excluded following Martinez-Boggio et al. (2025). Second, visits with CH_4_ flux below 15 g/d were excluded as a conservative threshold to remove likely measurement artifacts, such as visits with incomplete capture of eructation events. Third, statistical outliers were identified within each treatment as visits exceeding ± 3 SD from the treatment median for each gas (CH_4_, CO_2_, H_2_). This approach adapts the criterion of Martinez-Boggio et al. (2025) by stratifying by treatment to accommodate the Latin-square design. All visits passing these three filters were retained for the primary analysis regardless of the number of valid visits available per animal-period, provided at least one valid visit was recorded. Imposing a minimum visit threshold would selectively retain the most frequent GreenFeed users and bias results toward that subgroup, a limitation previously noted in GreenFeed-based emission studies (Hammond et al. 2016).

Emission yields (g gas/kg DMI) were calculated using a 7-d rolling average of DMI (including the day of gas measurement and the 6 preceding days) to account for the lag between feed intake and enteric fermentation. Emission intensity (g gas/kg ADG) was calculated at the period level by dividing mean gas production (g/d) by mean ADG (kg/d). Although DMI and ADG data were available for all 24 steers, these variables were analyzed using the same subset of observations as gas emissions (*n* = 57) to ensure that treatment effects on performance and emissions were evaluated on the same experimental unit, thereby allowing direct interpretation of emission yield and intensity metrics.

### Statistical analysis

All statistical analyses were conducted using R (version 4.5.1). Linear mixed-effects models were fitted using the lme4 (Bates et al. 2015), nlme (Pinheiro et al. 2023), lmerTest (Kuznetsova et al. 2017), and emmeans (Lenth and Piaskowski, 2026) packages.

A total of twelve response variables were evaluated, including gas production (CH_4_, CO_2_, H_2_; g/d), animal performance variables (DMI, ADG; kg/d; and FE; kg/kg), emission yield (g gas/kg DMI), and emission intensity (g gas/kg ADG). For each response variable, a structured set of candidate linear mixed-effects models (M1–M13) was evaluated to appropriately represent the replicated Latin square design, repeated measurements, and potential temporal and variance effects. All models included dietary treatment and experimental period as fixed effects. Random effects were specified as animal nested within square (1 | square/animal) to account for blocking by initial BW and repeated observations across experimental periods. Model structures were evaluated in three sequential categories: fixed-effects structure, residual correlation structure, and variance structure.

Models M1–M4 were random intercepts models differing in fixed-effects specification. Model M1 included additive fixed effects of treatment and period (Y ∼ treatment + period), whereas Model M2 included the treatment × period interaction (Y ∼ treatment × period). When evidence of carryover effects was detected via likelihood ratio test (*p* < 0.10), carryover-adjusted versions of these models were evaluated (M3 and M4), incorporating prior-period treatment as an additional fixed effect. Models M5–M10 extended the fixed-effects structures of M1 and M2 by incorporating within-animal residual correlation structures to account for repeated measures across periods. Specifically, compound symmetry correlation structures were evaluated in Models M5 (additive fixed effects) and M8 (interaction effects). First-order autoregressive correlation structures [AR(1)] were evaluated in Models M6 and M9, allowing correlation to decay across periods. Unstructured correlation matrices, allowing each pair of repeated observations to have unique covariance, were evaluated in Models M7 and M10. These models were fitted using the nlme framework. Models M11–M13 addressed potential heteroscedasticity in residual variance. Model M11 allowed residual variance to vary with fitted values using a power variance function (varPower). Model M12 allowed residual variances to differ by period, and Model M13 allowed residual variances to differ by treatment.

All candidate models were initially fitted using maximum likelihood estimation to allow comparison using Akaike’s Information Criterion (AIC). Model selection followed a dual-criteria approach. First, models were screened for convergence, absence of singularity, and acceptable residual diagnostics. Normality of residuals was assessed using the Shapiro-Wilk test, with *p* > 0.05 considered acceptable. Second, among models meeting diagnostic criteria, the model with the lowest AIC was selected as the final model for inference. Final selected models were refitted using restricted maximum likelihood for parameter estimation. When residual normality assumptions were violated on the original scale, logarithmic transformation of the response variable was evaluated. For log-transformed models, statistical inference was conducted on the transformed scale, while estimated marginal means were back-transformed and reported as geometric means for interpretability. Because the AIC-best model selected for each variable was not always an interaction-form model, we additionally tested the treatment × period interaction in each of the five interaction-form models defined in our model set (M2, M4, M8, M9, and M10) for every response variable.

Statistical significance was assessed using Type III ANOVA. Estimated marginal means were computed using the emmeans package (Lenth and Piaskowski 2026). Pairwise comparisons among treatments were conducted using Tukey-adjusted tests to control for multiple comparisons. Linear and quadratic dose-response trends across TBE inclusion levels were evaluated using orthogonal polynomial contrasts. Statistical significance was declared at *p* ≤ 0.05, and trends were discussed when 0.05 < *p* ≤ 0.10. Outlier detection was performed only at the raw data level. Treatment effects are reported as estimated marginal means ± highest SEM unless otherwise stated.

## Results

All 24 steers remained healthy and completed the trial. Three steers failed to voluntarily visit the GreenFeed system throughout the entire experiment and were excluded from the gas-emission analyses; an additional 6 animal-period combinations did not contribute a valid GreenFeed record during that specific period. This resulted in 57 of 72 possible observations for CH_4_ and CO_2_, and 56 for H_2_ due to one additional missing record. For performance variables (DMI, ADG, and feed efficiency), analyses were conducted on both the gas-emission cohort (*n* = 21 steers; 57 observations) and the full cohort of all 24 steers (72 observations). No significant carryover effects were detected for any response variable for which the carryover term could be tested (all *p* ≥ 0.203). For 5 variables (CH_4_ yield; H_2_ yield and intensity; DMI and FE in the gas cohort), the carryover term was confounded with the Latin square design (singular model fit) and could not be tested; therefore, final models did not include prior-period treatment as a covariate.

### Animal performance

Dry matter intake, ADG, and FE did not differ among dietary treatments in either the gas-emission cohort (*p* = 0.228, *p* = 0.751, and *p* = 0.954, respectively) or the full cohort (*p* = 0.451, *p* = 0.335, and *p* = 0.289, respectively; Table 2). In the gas-emission cohort, mean DMI was 14.1, 13.6, and 13.2 kg/d for the control, low TBE, and high TBE treatments, respectively. Average daily gain averaged 1.80, 1.70, and 1.73 kg/d for the control, low TBE, and high TBE treatments, respectively. Feed efficiency averaged 0.131, 0.133, and 0.133 kg/kg, respectively. The full cohort showed similar values (DMI: 13.6, 13.5, 13.3 kg/d; ADG: 1.82, 1.65, 1.79 kg/d; FE: 0.133, 0.123, 0.134 kg/kg). No treatment × period interactions were detected for DMI or ADG in any cohort, or for FE in the full cohort (*p* ≥ 0.142 across the five interaction-form models). For FE in the gas cohort, four of the five models supported no interaction (*p* ≥ 0.570), with one model (M10) yielding interaction (*p* = 0.013).

**Table 2:** Effect of tribromoethanol on dry matter intake (DMI), average daily gain (ADG), and feed efficiency (FE)^a^.

*Variable*	Model^a^	*n* ^c^	Control^d^	Low^d^	High^d^	% change relative to Control		SEM^e^	*P*-value^f^			
						Low	High		Treatment	Period	Linear	Quadratic
***DMI, kg/d***												
*** Gas steers***	M10	57	14.1	13.6	13.2	−3.55%	−6.38%	0.517	0.228	0.304	0.100	0.843
*** All steers***	M1	72	13.6	13.5	13.3	−0.735%	−2.21%	0.382	0.451	0.013	0.214	0.857
***ADG, kg/d***												
*** Gas steers***	M11	57	1.80	1.70	1.73	−5.56%	−3.89%	0.111	0.751	0.325	0.554	0.522
*** All steers***	M1	72	1.82	1.65	1.79	−9.34%	−1.65%	0.102	0.335	0.060	0.793	0.148
***FE, ADG/DMI***												
*** Gas steers***	M13	57	0.131	0.133	0.133	1.53%	1.53%	0.0122	0.954	0.463	0.784	0.881
*** All steers***	M6	72	0.133	0.123	0.134	−7.52%	0.752%	0.00709	0.289	0.131	0.959	0.117

a Estimated marginal means from linear mixed-effects models.

b Model codes: M1 = random intercepts (additive); M6 = AR(1) autocorrelation (additive); M10 = unstructured autocorrelation (interaction); M11 = variance proportional to fitted values; M13 = variance by treatment (additive).

c 3*n* = number of observations.

d Control = 0 mg 2,2,2-tribromoethanol (TBE)/kg DM; Low = 15 mg TBE/kg DM; High = 30 mg TBE/kg DM.

e SEM, Standard error of the mean.

f *P*-values: Treatment and Period from Type III ANOVA; Linear and Quadratic from orthogonal contrasts on TBE dose. Means with different letters differ at *p* < 0.05 (Tukey HSD).

**Figure 1:**
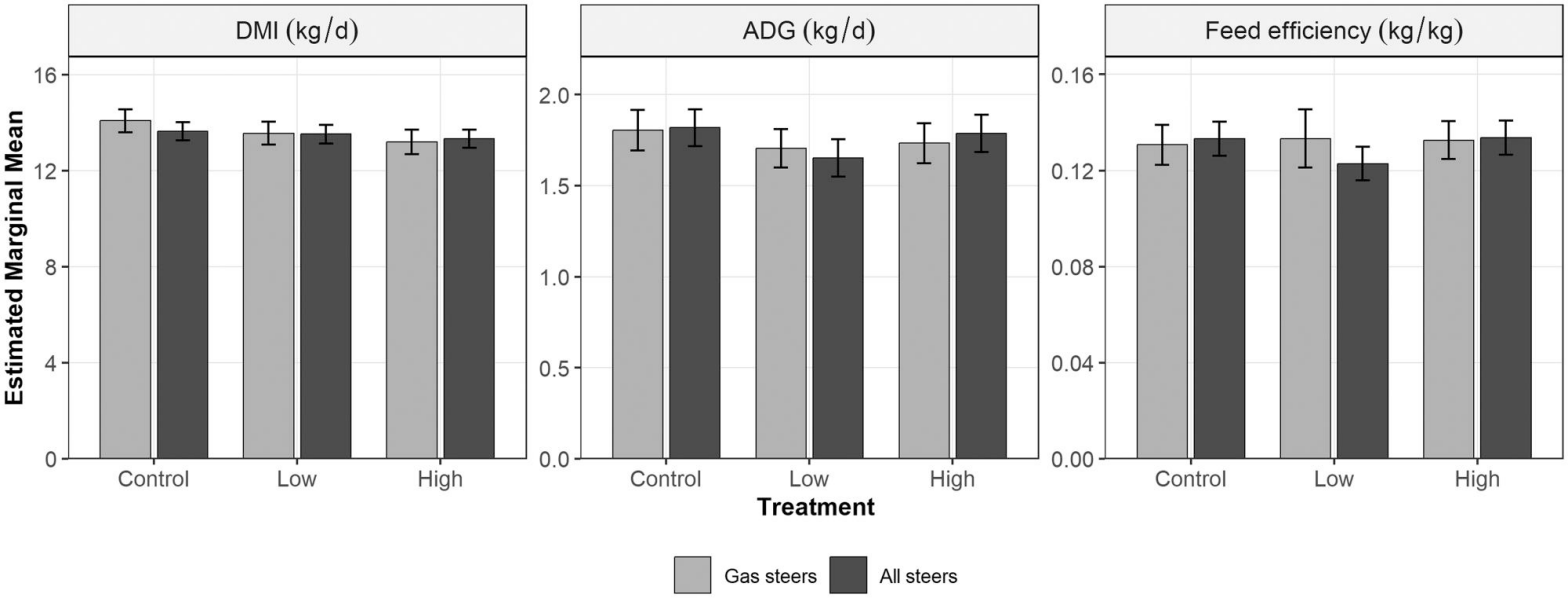
Dry matter intake (DMI), average daily gain (ADG), and feed efficiency (ADG/DMI) of beef steers supplemented with tribromoethanol (TBE) in a replicated 3 × 3 Latin square experimental design with 28-d experimental periods. Light bars represent the gas-emission cohort (*n* = 21 steers with valid GreenFeed measurements); dark bars represent the full cohort (*n* = 24 steers). Treatments were Control at 0 mg TBE/kg DM, Low TBE at 15 mg TBE/kg DM; or High TBE at 30 mg TBE/kg DM.

Body weight increased steadily over the course of the experiment for all treatments, with no evidence of treatment-related differences in growth (Fig. 1). These results indicate that supplementation with TBE did not adversely affect feed intake, growth performance, or feed efficiency under the conditions of this study. Neither linear nor quadratic contrasts of TBE dose were significant for DMI, ADG or FE (*p* ≥ 0.100).

### Enteric methane emissions

**Figure 2:**
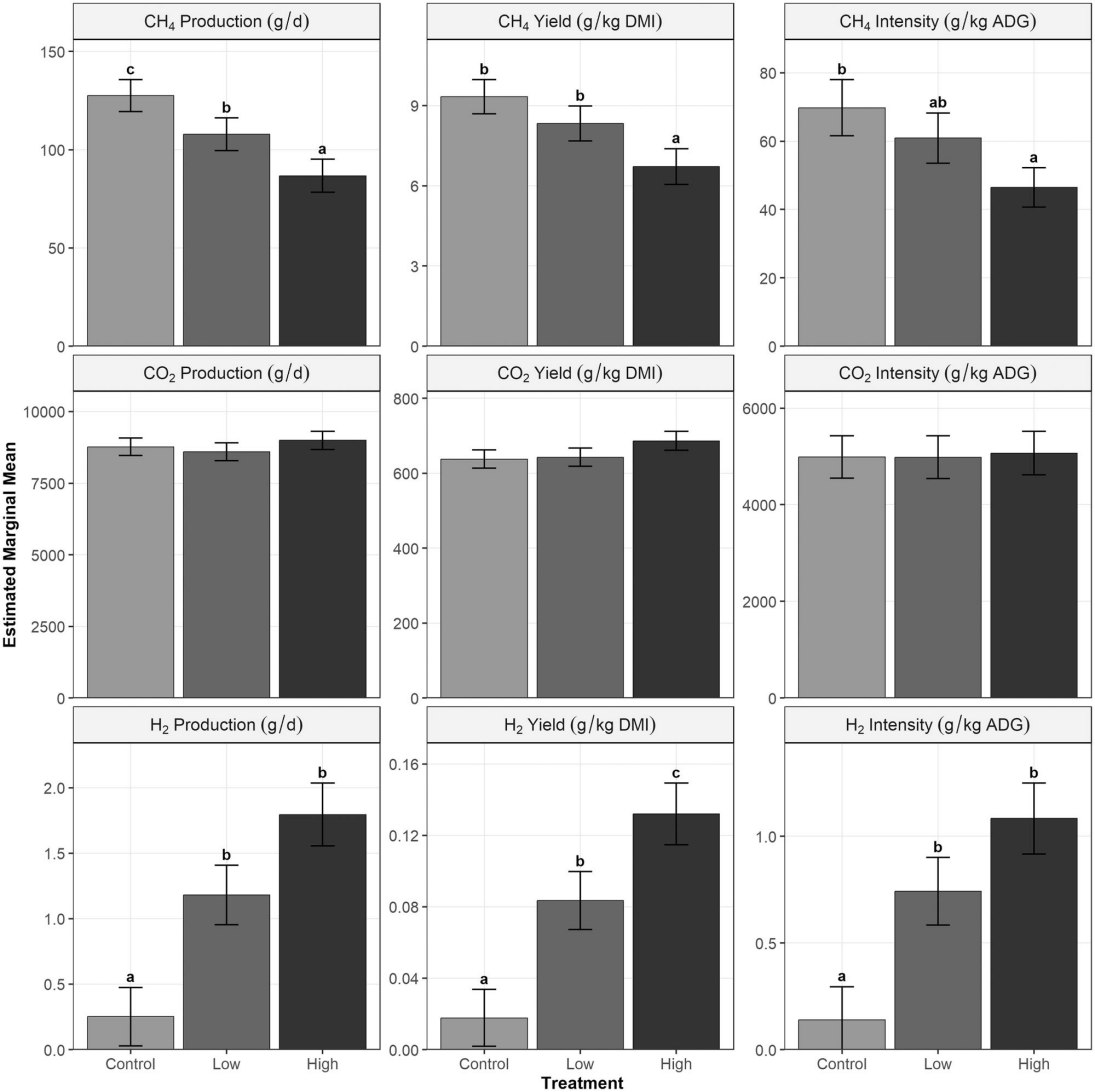
Methane (CH4), carbon dioxide (CO_2_) and hydrogen (H2) production, yield and intensity of beef steers supplemented with tribromoethanol (TBE) in a replicated 3 × 3 Latin square experimental design with 28-d experimental periods. Treatments were Control at 0 mg TBE/kg DM, Low TBE at 15 mg TBE/kg DM, and High TBE at 30 mg TBE/kg DM. Means with different superscript letters differ significantly (*p* < 0.05, Tukey HSD).

Dietary supplementation with TBE reduced daily CH_4_ production (*p* < 0.001; Table 3; Fig. 2). Mean CH_4_ production was 128 g/d for the control treatment, compared with 108 g/d and 86.8 g/d for the low and high TBE treatments, respectively. Relative to the control, CH_4_ production was reduced by 15.6% with the low TBE dose and by 32.2% with the high TBE dose. Methane yield was lower in cattle receiving the high TBE treatment compared with the control (*p* < 0.001), while no significant difference was observed between the control and low TBE treatments. Compared with the control treatment, CH_4_ yield decreased by 28.0% and 10.7% for the high and low TBE treatments, respectively (Table 3). Similar patterns were observed for intensity, as CH_4_ emissions per unit of animal growth reduced for cattle on the high TBE treatment relative to the control (*p* = 0.004), but not for those on the low TBE treatment. A 33.4% reduction in CH_4_ intensity was observed for cattle on the high TBE treatment, whereas a 12.8% reduction was observed for the low TBE treatment (Table 3).

**Table 3:** Effect of tribromoethanol on enteric gas emissions^a^.

*Variable*	Model^b^	*n* ^c^	Control^d^	Low^d^	High^d^	% Change relative to Control		SEM^e^	*P*-value^f^			
						Low	High		Treatment	Period	Linear	Quadratic
***Methane***												
*** Production (g/d)***	M9	57	128c	108b	86.8a	−15.6%	−32.2%	8.43	<0.001	<0.001	<0.001	0.909
*** Yield (g/kg DMI)***	M9	57	9.33b	8.33b	6.72a	−10.7%	−28.0%	0.669	<0.001	0.001	<0.001	0.567
*** Intensity (g/kg ADG)***	M1_(log)_	57	69.8b	60.9ab	46.5a	−12.8%	−33.4%	8.24	0.005	0.002	0.001	0.497
***Carbon Dioxide***												
*** Production (g/d)***	M9	57	8771	8593	8994	−2.03%	2.54%	318	0.338	0.005	0.405	0.212
*** Yield (g/kg DMI)***	M1	57	638	643	686	0.784%	7.52%	25.4	0.136	0.014	0.069	0.385
*** Intensity (g/kg ADG)***	M12_(log)_	57	4987	4984	5069	−0.0602%	1.64%	450	0.962	0.124	0.817	0.885
***Hydrogen***												
*** Production (g/d)***	M12	56	0.252a	1.18b	1.80b	368%	614%	0.240	<0.001	0.460	<0.001	0.499
*** Yield (g/kg DMI)***	M12	56	0.0178a	0.0835b	0.132c	369%	642%	0.0173	<0.001	0.594	<0.001	0.601
*** Intensity (g/kg ADG)***	M6	56	0.139a	0.743b	1.08b	435%	677%	0.166	<0.001	0.535	<0.001	0.398

a Estimated marginal means from linear mixed-effects models.

b Model codes: M1 = random intercepts (additive); M6 = AR(1) autocorrelation (additive); M9 = AR(1) autocorrelation (interaction); M12 = heterogeneous variance by period. (log) indicates log-transformed response; values are back-transformed.

c 3*n* = number of observations.

d Control = 0 mg 2,2,2-tribromoethanol (TBE)/kg DM; Low = 15 mg TBE/kg DM; High = 30 mg TBE/kg DM.

e SEM, Standard error of the mean.

f *P*-values: Treatment and Period from Type III ANOVA; Linear and Quadratic from orthogonal contrasts on TBE dose. Means with different letters differ at *p* < 0.05 (Tukey HSD).

Treatment × period interactions were detected for CH_4_ production (*p* = 0.015 to 0.032 across five interaction-form models) and yield (*p* = 0.036 to 0.081), driven by an attenuation of the High TBE effect in Period 3. Reductions relative to control for High TBE were −50.2%, −46.5%, −0.9% for CH_4_ production and −48.9%, −42.8%, 5.6% for CH_4_ yield in periods 1,2,3, respectively. The Low TBE effect was directionally consistent across periods, with reductions of −31.2%, −4.7%, and −10.5% for CH_4_ production and −12.7%, −9.2%, and −10.5% for CH_4_ yield. No treatment × period interaction was detected for CH_4_ intensity (*p* ≥ 0.451 across the five models). Linear contrasts were significant for CH_4_ production, yield, and intensity (*p* ≤ 0.001), with no significant quadratic component (*p* ≥ 0.497), indicating a linear dose-response relationship between TBE supplementation level and CH_4_ mitigation.

### Carbon dioxide and hydrogen emissions

Carbon dioxide emissions were not affected by dietary treatment (*p* > 0.05; Table 3; Fig. 2). Mean CO_2_ emissions did not differ among all treatments, indicating that overall fermentation activity and respiratory metabolism were not altered by TBE supplementation. In contrast, H_2_ emissions increased with increasing TBE dose (*p* < 0.001; Table 3; Fig. 2). Steers receiving TBE exhibited greater H_2_ emissions than the control treatment (Table 3). For CO_2_ production, evidence of a treatment × period interaction was borderline and model-dependent (*p* = 0.047–0.996 in interaction-form models). No treatment × period interactions were detected for CO_2_ yield (*p* ≥ 0.764), CO_2_ intensity (*p* ≥ 0.595), or any H_2_ response (production *p* ≥ 0.865; yield *p* ≥ 0.888; intensity *p* ≥ 0.817). Hydrogen emissions increased linearly with TBE dose (*p* < 0.001), with no significant quadratic effect (*p* ≥ 0.398). No linear or quadratic effects were detected for CO_2_ emissions (*p* ≥ 0.069).

Across all evaluated metrics, TBE supplementation resulted in linear dose-related reductions in enteric CH_4_ emissions without affecting DMI, ADG, FE or CO_2_ emissions. The increase in H_2_ emissions with TBE supplementation was consistent with inhibition of methanogenesis and redirection of metabolic H_2_.

## Discussion

The results of this study demonstrate that dietary supplementation with TBE reduced enteric CH_4_ emissions in beef cattle in a linear dose-dependent manner, while maintaining parameters such as feed intake, ADG, and feed efficiency. Mitigation strategies that suppress methanogenesis may risk compromising animal performance (e.g., oils depress DMI, Beauchemin and McGinn 2006), thereby reducing their practical value in commercial cattle production systems. In contrast, TBE supplementation reduced CH_4_ emissions with no statistical evidence of reduced intake or growth under the conditions of this short-term study, suggesting a favorable balance between environmental benefit and production efficiency. Interpretation of the performance responses should nonetheless consider two aspects of the study design. First, three steers were excluded from gas-related analyses due to failure to voluntarily visit the GreenFeed unit, reducing observations. These animals were healthy and their exclusion was unrelated to treatment. To address potential power loss, performance variables were analyzed using both the gas-emission cohort and the full cohort (Table 2), and results were consistent across both analyses. Second, the 28-d treatment periods, while well suited to capturing the CH_4_ response and consistent with established Latin square designs in this area, may have limited sensitivity for detecting subtle cumulative changes in intake behavior or growth trajectory. These considerations do not diminish the strength of the emissions findings but suggest that the null performance results be interpreted as strong preliminary evidence that TBE does not compromise intake or growth, with longer-term studies providing the opportunity to confirm this with greater statistical certainty.

Scientific literature examining TBE as a CH_4_ inhibitor remains limited. Recently, however, Fekri et al. (2026) demonstrated in vitro that TBE reduced predicted CH_4_ production by up to 28.4% without negatively affecting in vitro dry matter degradability or total volatile fatty acid production. To date, only two studies have evaluated TBE using an in vitro assay of CH_4_ production by rumen microorganisms, in which TBE was reported to be a potent inhibitor (Czerkawski and Breckenridge 1975; Fekri et al. 2026). To our knowledge, the present experiment is the first in vivo evaluation of TBE as an enteric CH_4_ mitigation strategy in cattle. Although the present study was not designed to directly assess microbial adaptation or other potential mechanisms underlying the period-specific changes observed in the High dose, demonstrating efficacy under in vivo conditions remains important because host interactions, feed intake dynamics, and ruminal microbial responses can differ substantially from those observed in vitro systems (Yáñez-Ruiz et al. 2016).

### Methane mitigation response to tribromoethanol

Supplementation with TBE reduced CH_4_ production by 15.6% at the lower inclusion rate of 15 mg/kg DM and by 32.2% at the higher inclusion rate of 30 mg/kg DM. Reductions were observed not only in absolute CH_4_ production but also in CH_4_ yield and CH_4_ intensity, confirming that the response was not driven by changes in intake or growth. The magnitude of reduction observed in the present in vivo study was comparable to the approximately 28% decrease in predicted CH_4_ production reported under in vitro conditions by Fekri et al. (2026), suggesting consistency between experimental systems.

The observed increases in H_2_ production with increasing TBE dose provide mechanistic support for the inhibition of methanogenesis. Methanogenic archaea serve as the primary H_2_ sink in the rumen by reducing CO_2_ to CH_4_ (Ungerfeld 2020), and when methanogenesis is inhibited, metabolic H_2_ accumulates unless alternative sinks are available. Similar increases in H_2_ emissions have been consistently reported for other direct inhibitors of MCR, including 3-NOP and bromoform (Haisan et al. 2014; Duin et al. 2016; Glasson et al. 2022). The lack of treatment effects on CO_2_ emissions further suggests that TBE selectively disrupted H_2_ utilization pathways rather than broadly suppressing rumen fermentation, which aligns with the targeted mode of action expected for halogenated CH_4_ analogues.

### Comparison with other feed additives targeting methanogenesis

Because tribromoethanol has not previously been evaluated in vivo as a CH_4_ mitigating feed additive, it is informative to contextualize the present findings alongside other additives that target rumen methanogenesis through similar biochemical pathways. In particular, 3-NOP and halogenated CH_4_ analogues such as bromoform represent well established and effective strategies that suppress CH_4_ formation by interfering with the terminal steps of methanogenesis, notably those involving MCR. Other halogenated compounds have also demonstrated methane-reducing effects in vivo. For example, bromochloromethane supplementation reduced enteric CH_4_ emissions in cattle (Tomkins et al. 2009). Collectively, these findings suggest that halogenated compounds may represent a broader class of CH_4_ inhibitors acting on archaeal methanogenesis pathways, although compound-specific efficacy, persistence, safety, and practical applicability may differ.

Across beef cattle studies, 3-NOP has repeatedly demonstrated its capacity to reduce enteric CH_4_ emissions, with reductions ranging from modest to substantial depending on dose, diet composition, and animal characteristics (Dijkstra et al. 2018; Yu et al. 2021). At higher inclusion rates, 3-NOP can achieve large and consistent reductions in CH_4_ emissions, while lower inclusion rates may yield more moderate responses.

Bromoform based strategies, whether delivered via macroalgae such as *Asparagopsis* or through synthetic formulations, represent another effective approach for suppressing enteric CH_4_ emissions. Numerous in vivo studies have demonstrated large reductions in CH_4_ production, often exceeding 80 to 95% under controlled conditions (e.g., Kinley et al. 2020; Roque et al. 2021). These responses are accompanied by marked increases in H_2_ emissions, reflecting strong inhibition of methanogenesis. While intake responses can vary depending on formulation, dose, and production context, bromoform is one of the most potent CH_4_ inhibitors identified to date and continues to receive considerable attention for its mitigation potential. The present results indicate that TBE produces a CH_4_ mitigation profile that aligns closely with these established additives in terms of direction and mechanism of response. As observed for both 3-NOP and bromoform, TBE supplementation reduced CH_4_ production while increasing H_2_ emissions and leaving CO_2_ emissions largely unchanged, consistent with selective inhibition of methanogenesis rather than broad suppression of rumen fermentation. Although the magnitude of CH_4_ reduction achieved with TBE in this study was moderate relative to high dose bromoform strategies, it was comparable to reductions reported for lower to moderate inclusion rates of 3-NOP in beef cattle (Dijkstra et al. 2018; Yu et al. 2021).

Halogenated methane analogues, including bromoform and chloroform, have been shown to share related biochemical targets within archaeal methanogenesis pathways, with the proposed mechanism involving interference with cobamide-dependent enzymatic steps, particularly those catalyzed by MCR (Wood et al. 1968; Tomkins et al. 2009; Duin et al. 2016). Although the specific mode of action of TBE has not been established, its structural similarity to other halogenated compounds suggests a comparable mechanism; however, this inference remains to be confirmed through mechanistic studies.

To further contextualize the magnitude of the CH_4_ mitigation response observed with TBE, it is useful to benchmark its efficacy against bromoform based feed additives using evidence synthesized across multiple in vivo studies. A recent comprehensive meta-analysis by Kebreab et al. (2025) evaluated the effects of bromoform containing seaweeds and synthetic bromoform based additives in both beef and dairy cattle and provides a robust reference for this comparison. Across 14 in vivo studies and 39 treatment comparisons, Kebreab et al. (2025) reported that at an average bromoform dose of approximately 28.3 mg bromoform/kg DM, CH_4_ production was reduced by 47.3%, CH_4_ yield by 43.3%, and CH_4_ intensity by 39.0%, with increasing dose associated with greater mitigation efficacy. These pooled estimates represent the central tendency of bromoform responses across a wide range of diets, cattle types, and delivery methods and therefore provide an appropriate basis for comparison. In the present study, supplementation with TBE at 30 mg/kg DM resulted in a CH_4_ production reduction of approximately 32%. Using the pooled CH_4_ production response from the bromoform meta-analysis as a reference point, a simple proportional scaling suggests that a CH_4_ reduction of this magnitude would correspond to a bromoform dose of approximately 19 mg/kg DM.

### Implications and future research needs

Although the CH_4_ reductions observed with TBE were moderate relative to those achieved with bromoform based technologies, the ability to reduce CH_4_ emissions without negatively affecting intake or growth highlights its potential as a mitigation tool in beef production systems. Long term studies are needed to assess the persistence of CH_4_ reductions, potential microbial adaptation, and any cumulative effects on animal health. In addition, evaluation of residue dynamics, toxicological safety, and regulatory feasibility will be essential before practical application can be considered. Further research should also evaluate TBE across a wider range of dietary conditions, production systems, and animal classes, as well as assess potential interactions with other mitigation strategies.

## Conclusion

Dietary supplementation with tribromoethanol reduced enteric CH_4_ production by 15.6%, CH_4_ yield by 10.7% and CH_4_ intensity by 12.8% at an inclusion level of 15 mg/kg DM. At the higher dose of 30 mg/kg DM, reductions increased to 32.2%, 28.0% and 33.4% for CH_4_ production, yield and intensity, respectively. Importantly, supplementation with TBE did not adversely affect DMI, growth performance, or FE. Taken together, these results demonstrate that TBE is among the effective CH_4_ inhibiting feed additives evaluated to date on a per unit intake basis, achieving substantial reductions in enteric CH_4_ emissions at relatively low inclusion rates while maintaining animal productivity. These findings provide the first in vivo evidence that tribromoethanol can suppress ruminal methanogenesis without compromising performance, indicating that it represents a promising candidate for greenhouse gas mitigation in cattle production systems.
